# Development and implementation of a formative instructional coaching program using the Teaching Practices Inventory within a health professions program

**DOI:** 10.1186/s12909-022-03616-z

**Published:** 2022-07-16

**Authors:** Amanda A. Olsen, Kathryn A. Morbitzer, Skye Zambrano, Jacqueline M. Zeeman, Adam M. Persky, Antonio Bush, Jacqueline E. McLaughlin

**Affiliations:** 1 School of Education, University of Texas at Arlington, Arlington, TX USA; 2 UNC Eshelman School of Pharmacy, University of North Carolina at Chapel Hill, Chapel Hill, USA; 3 American Medical Colleges, DC Washington, USA

**Keywords:** Teaching, Curriculum, Medical education, Pharmacy education, Instructional coaching, Peer observation

## Abstract

**Background:**

A growing body of literature describes teaching practices that are positively associated with student achievement. Observing, characterizing, and providing feedback on these teaching practices is a necessary, yet significant challenge to improving teaching quality. This study describes the design, implementation, and evaluation of an instructional coaching program created to provide formative feedback to instructors based on their use of evidence-based teaching practices.

**Methods:**

The program was designed for formative purposes utilizing an instrument adapted from the Teaching Practices Inventory. All faculty were invited to participate in the program on a voluntary basis when the program launched in Fall 2019. Program coaches included any School personnel who completed required training. Two rounds of instrument development were conducted with multiple observers and assessed using Krippendorff’s Alpha. The program was evaluated using an anonymous post-session survey.

**Results:**

Interrater reliability of the form improved over two rounds of piloting and no differences were found in scoring between trainees and education professionals. Seventeen observations were completed by nine coaches. Instructors indicated that feedback was practical, timely, specific, and collegial, suggesting that including student perspectives (e.g., focus groups, student course evaluations) in the coaching program might be helpful.

**Conclusions:**

Creating programs that emphasize and foster the use of evidence-based teaching are critical for health professions education. Additional research is needed to further develop coaching programs that ensure teaching practices in the health professions are optimizing student learning.

## Background

Utilizing teaching practices that effectively promote student development is critical to preparing the next generation of healthcare providers [1]. Despite widespread emphasis on evidence-based teaching, [2–6] health professions schools have faced considerable scrutiny concerning teaching quality [7, 8]. Studies indicate that, although health professions educators are experts in the content they teach, they rarely receive training on effective teaching practices [7, 8].

Observing, characterizing, and providing feedback on teaching practices is a necessary, yet significant challenge to improving teaching quality [9]. Debate surrounds the role and qualifications of the observer, characteristics and behaviors of the instructor, the observation process and criteria, and use of results [10–13] While student evaluations of teaching are a common strategy, research suggests that students lack crucial knowledge regarding how to appropriately evaluate effective teaching skills and that results are often subjective [11, 14–17]. Student evaluations can be biased against females, who are often rated according to personality and appearance, and faculty of color, who are subject to systemic biases like racial stereotyping [16, 17].

Peer observations are often recommended as additional evidentiary sources of teaching quality [11]. Two types of peer observations exist: summative observation and formative observation [18]. Summative observation, commonly known as “peer evaluation” or “peer review,” is designed to provide information that informs decision-making by the institution and is intended for use by others. These types of observations are routinely used by institutions for promotion, tenure/post-tenure review, reappointment, and merit awards, among others. As such, practically all health profession institutions have a summative peer observation process, and several have published on their programs [19–23].

Formative observation (also called peer feedback, peer observation, or peer coaching), is designed to provide feedback with the intent of personal use to improve the instructor’s quality of teaching. While summative observation is a necessary component for faculty, literature suggests that faculty also appreciate the availability of formative observation [24]. Institutions who have implemented formative observation programs have commented on their association with increased collegiality, acceptance of evidence-based changes in teaching pedagogy, and validation of good teaching practices [24–30]. However, formative observation processes can be resource and time intensive, with some concerned that only an expert in adult learning or curricular design could adequately conduct an observation [24–30].

Another core challenge of student and peer observations is instrument quality [31, 32]. Observation instruments are often limited by lack of specificity, poor psychometrics, and confusing directions [10]. Research suggests, for example, that observer ratings are often biased by preconceived notions of what constitutes effective teaching and the tendency to look for characteristics of themselves in the teaching of others [33].

Taken together, the complexities associated with observing and generating feedback about teaching practices can hinder programs as they strive to assess and improve instructional practice. The overarching purpose of this work was to develop a formative, evidence-based instructional coaching program (ICP) aimed at supporting and encouraging instructors to implement effective pedagogical strategies. This paper describes the development of the ICP, design of the ICP instrument, and initial evaluation of the ICP implementation.

## Methods

### Program development

**Fig. 1:**
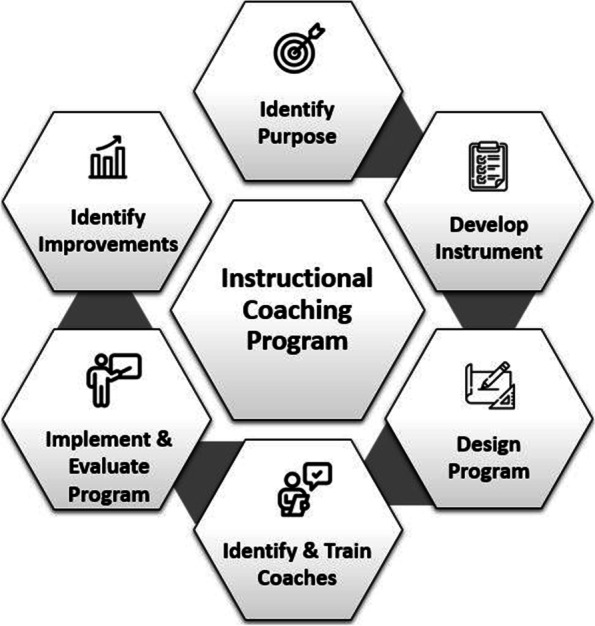
Process utilized to develop an instructional coaching program

The School’s Center for [BLINDED for REVIEW] at the [BLINDED for REVIEW] was asked by School leadership to develop a standardized peer observation process for faculty. While peer observations were already required at the School, observers used various instruments and criteria for the observations. The following steps describe the process utilized to develop, implement, and evaluate the ICP (Fig. 1). During each step, decisions were vetted with School leadership to ensure buy-in and support for the program.

#### Step 1: Establish a clear purpose

A development team was established that consisted of four faculty from varying tracks (i.e., fixed-term, tenure-track) and ranks (i.e., Assistant, Associate, Full) with experience and expertise in teaching across multiple degree programs at the School. The first decision in the development of the process was whether it should be formative, summative, or a combination of the two. In other words, what was the purpose and intended outcomes of the program? The development team agreed that a formative process was needed to provide instructors with evidence-based feedback about their teaching practices for professional growth and development. The team further agreed that combining formative and summative evaluations into one program could overshadow the value of the formative feedback and bias the evaluation provided by observers. Therefore, the team created two separate processes: one solely dedicated to summative purposes, and one solely dedicated to formative purposes. The formative process described below was termed the ICP.

#### Step 2: Develop a peer observation instrument

The ICP instrument was adapted from the Teaching Practices Inventory (TPI), which was developed by Wieman and Gilbert to reduce the subjectivity commonly associated with characterizing college teaching. 17 Each TPI item is evidence-based; in other words, each item was derived from research that demonstrated the extent to which different teaching practices were associated with student learning. 9 An item was assigned points based on the number of times the practice was used and its related effect size from the literature; for example, *Posed a question followed by a small group discussion* was scored as a “2” if the practice was used more than once and scored as a “0” if the practice was not used at all. 9.

Given the ability of the TPI to objectively and reliably characterize teaching practices, [9, 34–36] it was selected as the foundation for our coaching instrument. To focus the instrument on observable classroom teaching practices and enable the use of the instrument during a teaching observation, we identified 10 TPI items that aligned with the teaching philosophy of the School, which emphasizes learner-centered teaching pedagogies such as the flipped classroom model and problem-based learning [37]. The development of the instrument was led by three faculty members, who shared and vetted the instrument with various stakeholders, including academic leadership, course instructors, and educational researchers. The TPI items were piloted and evaluated, as described below. Feedback and edits were applied and the final ICP instrument contained the TPI items along with narrative feedback for instructors, including strategies to keep, strategies to start, strategies to stop (i.e. Keep, Start, Stop), and a summary with 1–3 prioritized evidence-based recommendations.

#### Step 3: Design a coaching program

The following program design was established with the hope of generating and providing feedback to instructors that promoted the awareness and uptake of evidence-based teaching practices. Ideally, two to three observers (also called “coaches”) would be available for each observation, with one serving as the lead coach (e.g., facilitating communication with instructor, collecting and aggregating observation data). Prior to the observation, the instructor and coaches would review the observation process, identify instructor needs/interests, and share any relevant materials. During the observation, the coaches would arrive (e.g., for in-person teaching) or log in virtually (e.g., for online teaching) and sit towards the back of the room or turn off video to minimize distractions; coaches would be instructed to not participate or intervene during an observation. The observations occurred in a typical teaching setting. Following the observation, the coaches would discuss their observations and create recommendations for the instructor. During a post-observation meeting with the instructor, the coaches would provide feedback, recommendations, and relevant handouts with supporting literature. At the conclusion of the post-observation meeting, instructors would be encouraged to watch the recording of their class session and reflect on their teaching practices and the feedback provided. Since the ICP was designed to provide formative feedback, ICP results would be provided only to the instructor, with the instructor allowed to share their own observation results at their discretion.

#### Step 4: Identify and train coaches

Based on the findings from the instrument evaluation, it was determined that, with appropriate training and a reliable observation instrument, any member within the School could serve as an observer (e.g., faculty, staff, trainees) for any classroom instructor. Participation as a coach was voluntary and was incentivized by recognizing the effort as service to the School during the annual review process. Coach training consisted of a pre-training assignment, which involved watching a class recording and completing the observation form. During the 60-minute in-person training session, coaches discussed how they marked each item and provided suggestions for improving the instrument.

#### Step 5: Implement and evaluate the coaching program

Since the focus of the program was growth and development, it was decided that participation would be voluntary for all School instructors. Reminders about the program were shared regularly via email and announcements at meetings. On an annual basis, the center worked with School leadership to identify any additional recommendations for ICP participants. As described in more detail below, data from ICP observation instruments and participant surveys were collected for each session.

#### Step 6. Identify improvements

Key to the success and sustainability of any program is the ability to adapt and improve. Feedback was solicited from instructors and coaches in an effort to identify opportunities for improvement of the instrument and program.

### ICP evaluation

To develop and refine the ICP instrument, two pilot evaluations were conducted. In the first round, 14 individuals completed the instrument while watching a recorded 50-minute biostatistics classroom session. The instrument asked for frequency counts representing the number of times each teaching practice was observed. The researchers converted the frequency counts to true scores, according to the quantification schema of the original TPI (Table 1). The total score for the instrument could range from 0 to 13. Based on results and feedback from round 1, five items were revised with minor wording changes. In round 2, the revised instrument was provided to 11 new pilot observers, who completed the same video observation. Observer position (e.g., faculty, postdoctoral fellow, student, staff) was collected from all pilot observers to examine differences in scores based on education position. Convenience sampling was used to identify and recruit all pilot observers, and all agreed to participate. Overall interrater reliability was calculated using Krippendorff’s Alpha [38] According to Krippendorff, [38] alpha levels above 0.67 are considered acceptable, with alpha levels above 0.80 considered ideal. To examine group differences, Mann Whitney U tests were applied for continuous items and chi-square tests for categorical items, with Fisher’s Exact Test used as needed. Nonparametric tests were utilized due to small sample sizes.

**Table 1:** Round 2 modified TPI questions and true score values

Question	Possible True Score
1. Asked for student questions OR paused for student questions [3 s minimum]^a^	1 if > 3
2. Used small group discussions or problem solving	1 if 1 2 if > 1
3. Asked students to predict results and then compared observations with predictions^b^	1 if ≥ 1
4. Posed a question followed by a small group discussion	2 if > 1
5. Used an audience response system	0
6. Followed an audience response system with a mini-lecture or discussion	1 if > 2
7. Percent of time spent lecturing	2 if 0–59% 1 if 60–79% 0 if 80–100%
8. Asked students to reflect on the lecture and/or their learning at the end of class^c^	1 if Yes
9. Expected students to view pre-class material and/or complete a related assignment before or at the start of class^d^	2 if Yes
10. Assessed student knowledge at the beginning of the class session	1 if Yes

Note: Original questions from round 1 stated below

^a^Asked for student questions OR paused for student questions for at least 3 s

^b^Asked students to record predicted results and then compared observations with predictions

^c^Asked student to reflect at the end of class

^d^Students expected to view pre-class material and complete related assignment before or at the start of class?

 To evaluate the ICP program, participating instructors were emailed an anonymous 3-item open text survey regarding their experience with the program after each ICP session. Descriptive statistics were used to summarize the number of faculty participants, observations, coaches, and time spent dedicated to the ICP. Qualitative data collected through open-ended responses on the participant survey were thematically coded by one researcher. Results are presented as median (range). This study was submitted to the University of North Carolina Institutional Review Board and the study did not constitute human subjects research as defined under United States federal regulations [45CFR 46.102 (d or f) and 21 CFR 56.102(c)(e)(l)]. Verbal consent was obtained.

## Results

In the ICP instrument evaluation, pilot observers were a combination of trainees (7 in round 1 and 4 in round 2) and education professionals (7 in round 1 and 7 in round 2). No differences were found in instrument scores between groups based on observer position. In addition, interrater reliability increased for categorical items from round 1 (α = 0.33) to round 2 (α = 0.60) and continuous data from round 1 (α = 0.61) to round 2 (α = 0.73).

Since the ICP launched in Fall 2019, we have completed 17 classroom observations of 16 individual instructors who requested coaching. Twelve observations were of a professional program course and five observations were of a graduate education course. Professional program courses included foundational courses and clinically focused courses. Fifteen instructors observed were faculty (8 assistant, 5 associate, 2 full) and one was a postdoctoral fellow. Two faculty were tenured, three were untenured on the tenure-track, and ten were fixed-term. Six faculty (four fixed-term assistant, one tenured associate, one fixed-term full) and three postdoctoral fellows completed training and served as a coach for at least one observation. Nineteen coaching hours were spent observing class sessions, eight hours for post-session coaches debrief, 8.5 h for post-session instructor debrief preparation (e.g., creating summary document), and 17 h for post-session instructor debrief.

Ten instructors (response rate = 63%) completed the optional, anonymous survey regarding their ICP experience. Participants found the feedback provided in the “Keep, Start, Stop” format with specific evidence-based recommendations particularly useful. As one participant shared, *I thought that the use of keep/start/stop was very helpful. The feedback was practical, timely, and specific and I plan to incorporate into my teaching right away*. Common areas of teaching that peer observers highlighted include wait time after prompting for questions, use of small group discussions, summarizing take-home points at the end of the class session, and assessments within pre-class materials. Participants also appreciated the opportunity to connect with other instructors as coaches and have two-way dialogue, which as one participant described, *was an opportunity to discuss what my [teaching] concerns were and to get feedback on if they’re actual things I need to work on or just misconceptions I have about teaching and learning.*

When asked for suggestions for improvement of the ICP, three participants suggested including a second observation as part of the ICP. Additional suggestions included having a pre-meeting with the instructor before the session being observed, having the instructor provide a written reflection on the class ahead of the session, and providing the instructor with key papers that support the recommendations provided. When asked to select additional aspects of teaching that they would find helpful in the ICP, participants most frequently selected student perspectives (e.g., coach-led focus groups with students from class) [*n* = 6], course evaluation review (e.g., discussion of most recent student course evaluation results) [*n* = 6], and pre-class review (e.g., organization, clarity, length of materials) [*n* = 5].

## Discussion

Conducting peer observations of teaching is a complex undertaking that can be influenced by a number of factors, including observer bias and instrument quality [26, 39]. By design, the TPI was developed to address these issues; however, it has largely been used as a self-reflection instrument since its release [9]. This study explored the utility of the instrument for observing classroom instruction as part of a formative coaching program. Our findings advance previous research on the TPI, namely by (1) expanding the use of the TPI to formative peer observations and (2) demonstrating that an adapted TPI can be utilized by various observers to generate feedback aimed at supporting educator development.

Studies suggest that health professions schools, and higher education in general, fall short of their potential to assess the teaching practices of their educators [11]. Students, peer-colleagues, and administrators are often not trained in effective teaching practices [40–42]. Continued development and use of instruments that limit subjectivity and promote evidence-based teaching practices is crucial for improving health professions curricula. Although other instruments exist, such as the Classroom Observation Protocol for Undergraduate STEM (COPUS) [43] and the Practice Observation Rubric to Assess Active Learning (PORTAAL), [44] the TPI provides a relatively simple, frequency-based system for characterizing research-based teaching practices with little to no training.

Summative peer observations are frequently utilized for personnel and award decisions, yet their usefulness for individual faculty growth and development are limited. The ICP provides a framework and process for providing formative feedback to instructors and engaging in discussion that emphasizes teaching practices known to promote student achievement. One strength of our program was the utilization of the post-observation meeting with the instructor and coaches. This allowed dedicated time for the instructor to reflect on their class session, receive feedback and recommendations from the coaches, and discuss potential ideas and next steps for their teaching. Since mentoring can also play an important role in the development of educators, consideration should be given to the potential role of mentors in instructional coaching [45].

The number of coaches trained for the ICP also alleviated the time and effort required by any one person. This was possible as the use of the adapted TPI as the observation form and coaching training program opened availability for any School member to serve as a coach. We also believe that keeping ICP feedback confidential (i.e. providing results only to the instructor) fostered a trusting environment with the main emphasis on providing constructive feedback and specific recommendations for improvement.

Despite the promising results of this study, many questions about the TPI and formative coaching programs remain. While the instrument and ICP described in this study may hold promise for providing more objective and reliable evidence of observable teaching practice for the purpose of improving teaching, it may not be appropriate for all learning environments. Namely, the TPI was developed using literature for classroom-based learning in which the instructor played an active role in facilitating learning. As such, learning environments in which the instructor is less active (e.g. small group facilitation) or learning is experiential (e.g. clinical rotations) may not be well-suited for the TPI.

There are several noteworthy limitations to this work. First, the results were drawn from a small, convenience sample, which may reduce the generalizability of these findings. The sample size also influenced the type of data analysis that was completed and statistical power. Second, the interrater reliability results while piloting the adapted TPI were lower than expected, suggesting that individuals may have interpreted items differently. While interrater reliability improved from round 1 to round 2, future efforts may focus on further refinement to increase interrater reliability. Lastly, the long-term impact of the ICP is unknown, including the percentage of recommendations implemented within an instructor’s own teaching practices and ICP coach satisfaction. Since the ICP is ongoing, more research will be done to advance this work.

## Conclusions

Understanding the effectiveness of teaching practices in the health professions is a complex undertaking. Common strategies for peer observation of teaching are often limited by observer bias, poor instrumentation, and a lack of focus on practices known to be associated with student learning. Further, educator development during peer observation is often hindered by the pressures and stakes associated with summative use of the results. The formative instrument and program developed in this study focused on observable classroom teaching practices and feedback that can enhance educator understanding of evidence-based teaching. The tool and strategies described could be applied to a variety of health professions educational settings (e.g., pharmacy, medicine, nursing, social work) with the goal of informing teaching practices, enhancing student learning, and ultimately improving patient outcomes. Additional research is needed to further develop the instrument and program to ensure that teaching practices in the health professions are preparing students for healthcare practice.

## Data Availability

The datasets generated and analyzed during the current study are not publicly available due to the small sample size and possibility of compromising anonymity/individual privacy; however, data may be made available from the corresponding author on reasonable request.
